# Relationship between blood pressure levels and ischemic stroke, myocardial infarction, and mortality in very elderly patients taking antihypertensives: a nationwide population-based cohort study

**DOI:** 10.1186/s12877-021-02570-7

**Published:** 2021-11-02

**Authors:** Myung-Sun Song, Yeon joo Choi, Hyunjin Kim, Myung Ji Nam, Chung-woo Lee, Kyungdo Han, Jin-Hyung Jung, Yong-Gyu Park, Do-Hoon Kim, Joo-Hyun Park

**Affiliations:** 1 Department of Ophthalmology, College of Medicine, Korea University Anam Hospital, Seoul, Republic of Korea; 2 Department of Family Medicine, Korea University Ansan Hospital, Korea University College of Medicine, 123, Jeokgeum-ro, Danwon-gu, Gyeonggi-do Ansan, Republic of Korea; 3 Department of Statistics and Actuarial Science, Soongsil University, Seoul, Republic of Korea; 4 Department of Biostatistics, College of Medicine, The Catholic University of Korea, Seoul, Republic of Korea

**Keywords:** Elderly, Hypertension, Blood pressure, Ischemic stroke, Myocardial infarction, Mortality

## Abstract

**Background:**

In the very elderly, “the lower the better” hypothesis has constantly been contradicted by randomized control trials and various cohort studies, but inconsistency in results led to unclear blood pressure treatment targets. This study aimed to assess the relationship between baseline blood pressure (BP) and ischemic stroke, myocardial infarction, and all-cause mortality in very elderly people treated for hypertension.

**Methods:**

This large population-based retrospective cohort study was based on the national claims database of the Korean National Health Insurance System, which covers the entire Korean population. 374,250 participants aged ≥ 75 years taking antihypertensive agents were recruited, excluding patients with a history of previous ischemic stroke or myocardial infarction.

**Results:**

Systolic BP (SBP) followed a J curve for ischemic stroke and a U curve for all-cause mortality, with nadir ranges of 120 to 129 mmHg and 140 to 149 mmHg, respectively. While increasing diastolic BP (DBP) generally resulted in higher HRs for ischemic stroke, HRs for myocardial infarction and all-cause mortality significantly increased only when DBP was ≥ 80 mmHg and ≥ 90 mmHg, respectively. The SBP/DBP combination analysis showed that even with SBP < 130 mmHg, higher DBP ≥ 90 mmHg had higher HRs for all three outcomes compared to the reference group (130 to 149 / < 80 mmHg).

**Conclusions:**

There were no further benefits or even harm below certain BP levels for ischemic stroke, myocardial infarction, and all-cause mortality in very elderly hypertensive patients.

## Background

Hypertension is a major risk factor for cardiovascular disease and stroke, and nearly 9.4 million people around the globe die from its complications annually [1]. Hypertension prevalence increases with age [2], and with the rapid aging of societies, particularly the oldest old being the fastest growing portion [3], managing hypertension among the very elderly becomes key to controlling the impending global health crisis.

Like the younger population [4], very elderly people also benefit from antihypertensive treatment [5], but the ongoing debate on the optimal therapeutic blood pressure target has yet to be settled. In the HYVET trial, patients 80 years or older, when treated to achieve a goal blood pressure (BP) of 150/80 mmHg, experienced significant reduction in stroke mortality, heart failure, and all-cause mortality [5]. On the other hand, two Japanese trials showed there was no additional benefit of further lowering systolic BP (SBP) below 140 mmHg [6, 7]. Furthermore, low SBP and excessive BP reduction in the very elderly have been reported to be associated with higher risk [8–13]. The J-curve observed in a Unites States (US) veteran cohort [8] and in the INVEST trial sub-study [9] illustrates an initial decline in the hazard ratio (HR) in octogenarians as SBP is lowered, but below 140 mmHg cardiovascular outcomes [9] and total mortality [8, 9] increase with an additional SBP decrease. Accordingly, a recent cohort of octogenarians from the United Kingdom (UK) demonstrated the lowest mortality rate at SBP of 140-159 mmHg, with increasingly greater HRs for lower SBP ranges [14]. Conversely, in the SPRINT trial subgroup analysis of individuals 75 years or older, intensive treatment with a goal of SBP < 120 mmHg resulted in significantly lower rates of cardiovascular events, stroke, and all-cause mortality [15]. This led to the American College of Cardiology/American Heart Association (ACC/AHA) hypertension treatment guidelines to be revised to a target BP of 130/80 mmHg even for the elderly [16], raising some concerns among experts on how caution must be taken with this new target.

To address the inconsistency of results on the effects of blood pressure in the very elderly, this study used the National Health Insurance Service (NHIS) database, which covers the entire Korean population. We evaluated Koreans 75 years or older treated for hypertension, examining the association between baseline blood pressure and ischemic stroke, myocardial infarction (MI), and all-cause death. Beyond looking at SBP and DBP individually, we also analyzed SBP/DBP combination groups to examine the real-world situation where the two parameters are controlled not separately, but rather together and may interact. The importance of this study lies in the large population size of nationwide claims data and its focus on the clinically relevant population undergoing antihypertensive treatment. This study will also add reliable evidence to the limited knowledge currently available for hypertension in the very elderly Asian population. Moreover, beyond all-cause mortality which has been the key focus of previous large population cohort studies, we also addressed ischemic stroke and MI, which are representative of the coronary and cerebrovascular complications of hypertension. Lastly, the SBP/DBP combination analysis may provide evidence to further our understanding of BP parameters and suggest potential aspects to consider for managing BP in the real-world clinical setting.

## Methods

### Data source and establishment of the cohort

In this retrospective cohort, we used the national claims database established by the NHIS of Korea. In Korea, the entire population has mandatory coverage under NHIS, and every two years they are recommended to receive a medical checkup [17]. The NHIS stores data including demographics, diagnoses using the International Classification of Disease, Tenth Revision (ICD-10) codes, procedures, and prescriptions [18]. All Koreans ≥ 75 years of age who underwent medical checkup between January 1, 2009 and December 31, 2012, were recruited. From this population, the clinically relevant population receiving antihypertensive treatment was extracted using previous diagnosis based on ICD-10 codes (I10-13, I15), along with medication history of one or more antihypertensive drugs. To identify patients with a first occurrence of events, we excluded those with a prior history of ischemic stroke and MI as defined by a recent 5-year diagnosis by ICD codes (I-63, 64 and I-21, 22). A total of 374,250 subjects were eligible for the cohort, and were followed for MI, ischemic stroke, and all-cause death until December 31, 2016. As this was a retrospective study and all data were anonymous and de-personalized, the need for individual consent was not applicable. All methods were performed in accordance with the relevant guidelines and regulations, including the Declaration of Helsinki. This study was approved by the Institutional Review Board (IRB) of the Korea University Ansan Hospital (2017AS0437).

### Definitions

Participants’ blood pressure measured at their initial medical checkup between 2009 and 2012 was set as the baseline BP. It was recommended to measure blood pressure on the right arm after resting for 5 min or more in a sitting position using a standard mercury sphygmomanometer. In addition, it was recommended to measure systolic blood pressure and diastolic blood pressure three times at 5-minute intervals if possible, and report the average value of the second and third measurements.

Rather than considering blood pressure measurements as continuous variables, we divided them into possible subgroups to find the optimal nadir for blood pressure control, and explored their relevance with key prognostic indicators. Therefore, Baseline BP was further stratified into eight subgroups in increments of 10 mmHg for SBP (< 110, 110–119, 120–129, 130–139, 140–149, 150–159, 160–169, ≥ 170 mmHg) and 5 mmHg for DBP (< 70, 70–74, 75–79, 80–84, 85–89, 90–94, 95–99, ≥ 100 mmHg).

For SBP/DBP combination analysis, we set the classification points as 130 and 150 mmHg for SBP, and 80 and 90 mmHg for DBP. Through this we aimed to predict the possible real-world results of adapting the new ACC/AHA target of 130/80 mmHg, compared to the previous target of 150/90 mmHg.

The outcome measures of this study were the first occurrence of ischemic stroke, MI, and death from any cause. Ischemic stroke was identified using ICD-10 codes (I63, I64) along with claims for one or more brain imaging studies (brain computed tomography [CT] or magnetic resonance imaging [MRI]), both prescribed during hospitalization. MI was defined by ICD-10-CM codes (I21, I22) recorded during hospitalization. These definitions had been validated in previous studies using NHIS data [19, 20].

The following covariate data, considered as major risk factors for stroke or MI and also major comorbidities of hypertension, were collected and classified to establish the baseline characteristics for the cohort: age (75 to 79 years, ≥ 80 years), sex, smoking (non-smoker, less than 100 cigarettes in a lifetime; ex-smoker, more than 100 cigarettes but former smoker; current smoker, more than 100 cigarettes and current smoker), alcohol intake (none; mild, less than 30 g/day; heavy, more than 30 g/day), physical activity (no regular exercise; regular exercise, 3 or more days of intense 20 min daily workouts, or 5 or more days of moderate 30 min daily workouts, weekly), yearly income (lowest quartile, remaining quartiles), body mass index (BMI) and obesity (BMI ≥ 25 kg/m^2^), waist circumference and abdominal obesity ( ≥ 90 cm for males and ≥ 85 cm for females). Lifestyle data were based on answers provided by subjects in standardized questionnaires conducted upon their national medical checkup.

Diabetes was determined based on fasting blood glucose levels of ≥ 126 mg/dL measured during the medical checkup or based on ICD-10-CM codes (E11-E14) along with registered anti-diabetic drug prescriptions, and dyslipidemia with total cholesterol levels ≥ 240 mg/dL, or ICD-10-CM code E78 and lipid lowering drug prescriptions. Chronic kidney disease (CKD) was identified by eGFR calculation below 60 mL/min/1.73m^2^. The operational definitions of these comorbidities have also been validated in previous cohorts [18, 20].

### Statistical Analysis

Baseline characteristics are presented as means with standard deviation for continuous variables and numbers and percentages for categorical variables. Annual incidence rates (IR) were calculated as the number of events per 1000 person-years. Cox proportional hazard models were used to estimate HR and 95 % confidence intervals (CI) to analyze association between baseline blood pressure subgroups and cardiovascular and mortality outcomes. Covariates (age, sex, lifestyle factors, and comorbidities) were entered into the models for adjustment. Additional analysis according to SBP/DBP combination groups was performed. Two-tailed P-values < 0.05 were considered statistically significant. Statistical analyses were conducted using SAS version 9.4 (SAS Institute Inc, Cary, NC, USA).

## Results

**Fig. 1:**
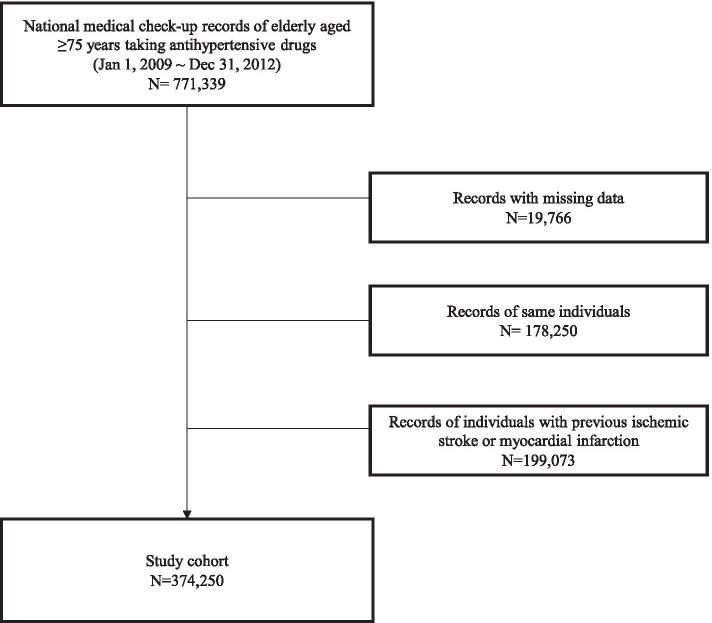
Flowchart of cohort selection. A total of 374 250 subjects were eligible for the cohort, and were followed for MI, ischemic stroke, and all-cause death until December 31, 2016

This study included 374,250 hypertension treated Koreans 75 years or older (Fig. 1), whose baseline characteristics by outcome measure are shown in Table 1. During mean follow-up of 5.6 years, 28,621 patients experienced ischemic stroke, 14,683 had MI, and 74,115 patients died. Generally, patients with cardiovascular event incidence had higher rates of CVD risk factors. Compared to participants with no events, those with ischemic stroke and MI incidence had significantly higher baseline blood pressure levels, were older, more likely to be male, be current smokers, be physically inactive, have diabetes, dyslipidemia, and chronic kidney disease. Alcohol consumption showed different trends for ischemic stroke and MI. The no event groups had higher rates of abdominal obesity and obesity. Interestingly, in regards to mortality, people who died were less likely to have dyslipidemia.

**Table 1:** Baseline Characteristics of the Study Cohort by Outcome Measure

	Ischemic Stroke			MI			Death		
	No	Yes	P-value*	No	Yes	P-value*	No	Yes	P-value*
n	345 629	28 621		359 567	14 683		300 135	74 115	
Mean (SD) age† (years)	78.6 (3.5)	78.9 (3.5)	< 0.01	78.6 (3.5)	79.1 (3.7)	< 0.01	78.2 (3.1)	80.3 (4.4)	< 0.01
Age group (%)			< 0.01			< 0.01			< 0.01
75-79 years	93.2	92.4		93.2	91.2		95.5	83.8	
≥80 years	6.8	7.6		6.8	8.8		4.5	16.3	
Male (%)	37.1	41.9	< 0.01	37.4	39.9	< 0.01	34.7	49.0	< 0.01
Smoking (%)			< 0.01			< 0.01			< 0.01
No	78.9	75.7		78.8	75.3		80.5	71.2	
Ex-smoker	13.6	13.7		13.6	13.3		12.9	16.4	
Current	7.5	10.6		7.6	11.4		6.6	12.4	
Drinking (%)			< 0.01			< 0.01			< 0.01
No	82.2	80.3		82.0	83.4		82.4	80.6	
Mild	15.5	16.7		15.7	14.2		15.5	16.2	
Heavy	2.3	3.0		2.4	2.4		2.2	3.2	
Regular exercise (%)	30.1	27.7	< 0.01	30.1	27.1	< 0.01	31.1	25.1	< 0.01
Lowest quartile income (%)	19.8	20.1	0.17	19.7	21.7	< 0.01	19.3	21.8	< 0.01
DM (%)	25.7	30.6	< 0.01	25.9	30.5	< 0.01	25.0	30.5	< 0.01
CKD (%)	25.1	28.8	< 0.01	25.2	30.0	< 0.01	23.9	31.6	< 0.01
Dyslipidemia (%)	33.9	34.4	0.1	33.8	37.2	< 0.01	35.4	27.9	< 0.01
Abdominal obesity (%)	53.2	52.1	< 0.01	53.2	52.0	< 0.01	55.4	43.9	< 0.01
Obesity (%)	34.8	33.7	< 0.01	34.8	32.5	< 0.01	37.0	25.8	< 0.01
Mean (SD) BMI^a^ (kg/m^2^)	23.9 (3.2)	23.8 (3.2)	< 0.01	23.9 (3.2)	23.6 (3.3)	< 0.01	24.1 (3.2)	22.9 (3.4)	< 0.01
Mean (SD) SBP^a^ (mmHg)	134.6 (16.6)	135.6 (17.4)	< 0.01	134.6 (16.6)	135.1 (17.3)	< 0.01	134.8 (16.4)	134.2 (17.7)	< 0.01
Mean (SD) DBP^a^ (mmHg)	79.1 (10.3)	80 (10.6)	< 0.01	79.1 (10.3)	79.7 (10.6)	< 0.01	79.2 (10.2)	79.2 (10.8)	0.81

*P-values were calculated using the t-test for continuous variables and chi-squared test for categorical variables. ^a^Continuous variables were expressed as mean (standard deviation). Abbreviations: MI, myocardial infarction; DM, diabetes mellitus; CKD, chronic kidney disease; BMI, body mass index; SBP, systolic blood pressure; DBP, diastolic blood pressure

**Fig. 2:**
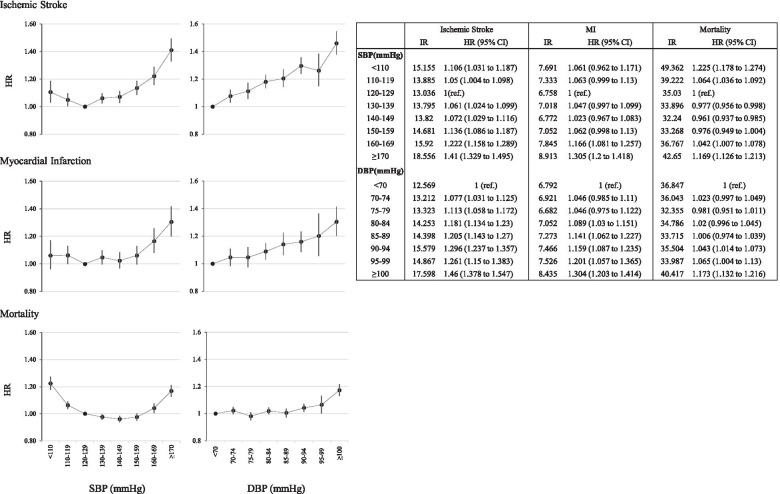
Hazard ratios (HR) for ischemic stroke, myocardial infarction (MI), and mortality according to the systolic blood pressure (SBP) and diastolic blood pressure (DBP) group. The data are adjusted for age, sex, body mass index (BMI), smoking/ drinking/exercise/income status, and the presence of diabetes mellitus (DM), dyslipidemia, and chronic kidney disease (CKD). The 95% confidence (CI) intervals are shown as vertical lines with the HRs

Cox regression analysis of adjusted HRs by SBP and DBP are shown in Fig. 2. The reference groups were SBP 120 to 129 mmHg and DBP < 70 mmHg. All three outcomes measures showed highest HRs and IRs with SBP ≥ 170 mmHg or DBP ≥ 100 mmHg, except for all-cause death with highest risk when SBP < 110 mmHg. HR by SBP followed a J-curve pattern for ischemic stroke, and a U-shaped pattern for all-cause death. The nadir SBP ranges were 120 to 129 mmHg (HR 1, reference group) and 140 to 149 mmHg (HR 0.961; 95 % CI 0.937 to 0.985) for ischemic stroke and all-cause death, respectively. For MI, SBP < 160 mmHg was a relatively homogenous group and HR significantly increased only once SBP ≥ 160 mmHg. Meanwhile, increasing DBP generally showed higher HRs for ischemic stroke. For MI and all-cause death, DBP up to 80 mmHg and 90 mmHg did not show any significant differences from the reference group, and HR significantly increased when DBP was ≥ 80 mmHg or ≥ 90 mmHg, respectively.

**Fig. 3:**
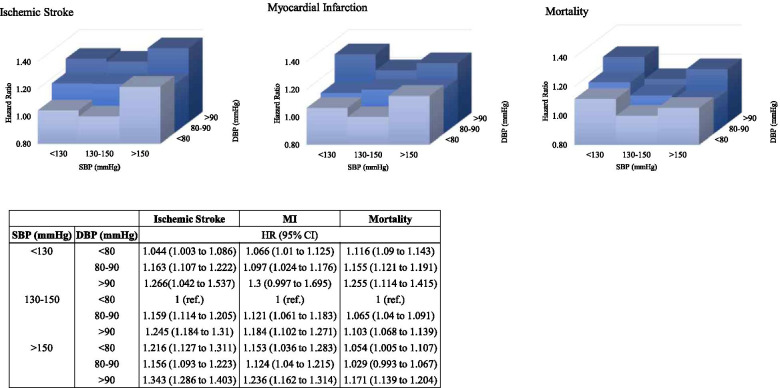
Hazard ratios (HR) for ischemic stroke, myocardial infarction, and mortality according to the systolic blood pressure/ diastolic blood pressure (SBP/DBP) combination group. The data are adjusted for age, sex, body mass index (BMI), smoking/drinking/exercise/income status, and the presence of diabetes mellitus (DM), dyslipidemia, and chronic kidney disease (CKD)

In the SBP/DBP combination analysis, HRs stratified by SBP/DBP groups were calculated (Fig. 3). The reference group was SBP 130 to 149 mmHg/DBP < 80 mmHg. For each SBP group, higher DBP groups yielded higher HRs for all three outcomes, except when SBP ≥ 150 mmHg, DBP < 80 mmHg had higher HRs than when DBP was 80 to 89 mmHg, also for all three outcomes. Regardless of SBP control, patients with the highest DBPs ≥ 90 mmHg had higher HRs compared to the reference group. While there was no additional consistent trend in ischemic stroke and MI, in terms of all-cause death, within the same DBP group, the highest HRs lay in the SBP groups < 130 mmHg.

## Discussion

In this nationwide cohort of 374,250 very old Koreans treated for hypertension, our data showed that there was a relationship between baseline blood pressure and ischemic stroke, MI, and all-cause death, after adjusting for age, sex, BMI, smoking, drinking, exercise, income status, diabetes mellitus, dyslipidemia, and chronic kidney disease.

SBP followed a J-curve for ischemic stroke and a U curve for all-cause death, with nadir ranges of 120 to 129 mmHg and 140 to 149 mmHg, respectively. However, the absolute increase in stroke or death at lower blood pressure was relatively small, based on the annual incidence of the key endpoint.

The HR for MI was similar to the reference group when SBP was < 160 mmHg, but was significantly increased when SBP was ≥ 160 mmHg. While a high DBP yielded a higher risk for MI and death, DBPs below 80 mmHg and 90 mmHg, respectively, had similar risks compared to the reference groups. The SBP/DBP combination analysis revealed that even with treatment SBP < 130 mmHg, higher DBP ≥ 90 mmHg had a higher HR compared to the reference group, for all three outcome measures. Within the same DBP level, the death hazard was higher for SBP < 130 mmHg than when SBP was 130 to 149 mmHg.

### Interpretation and comparison with other studies

The most important issues regarding BP control in the very elderly are: (1) is the lower the better, or is there a cutoff point below which there is no benefit or even harm? and (2) if there is a cutoff point, can it be defined? Our findings imply that very low SBP may not further reduce the risk of ischemic stroke, MI, and all-cause death in the very elderly. SBP below nadirs for ischemic stroke and death showed higher risk, and for MI, SBP < 160 mmHg represented a homogenous group with no significant difference from the reference group. Similarly, for DBP in MI and all-cause death, a DBP < 80 mmHg and < 90 mmHg had a relatively similar risk as the reference group with DBP < 70 mmHg, suggesting there may be no further benefits below a certain DBP level. These results are in agreement with findings from previous randomized controlled trials in the very elderly, indicating that lowering BP has cardiovascular and survival benefits [5], but below certain levels may not be beneficial [6, 21]. The HYVET trial showed that targeting BP levels below 150/80 mmHg reduced stroke mortality and total mortality [5], but the JATOS [6] and VALISH [21] trials found there were no further benefits below SBP < 140 mmHg.

Observational studies also suggest that there may be a cutoff point for cardiovascular benefits. In the INVEST sub-study of elderly patients aged ≥ 80 years, the primary outcome of total mortality, nonfatal stroke, or nonfatal MI followed J-curves for both SBP and DBP, with nadirs at 140 mmHg and 70 mmHg, respectively [9]. Moreover, the CLARIFY cohort showed that for elderly aged > 75 years, the HR of primary composite outcome of cardiovascular death, MI, and stroke was significantly higher with an SBP < 120 mmHg or DBP < 70 mmHg, than for the reference SBP 120 to 129 mmHg or DBP 70 to 79 mmHg [22].

 On the contrary, results of the most recent SPRINT trial and the ACC/AHA guideline revisions seem to support “the lower the better” hypothesis. In the SPRINT trial, even in elderly patients aged ≥ 75 years, there were significant mortality benefits and coronary, cerebrovascular event reductions with a target SBP of 120 mmHg [15]. However, it should be taken into consideration that this trial excluded patients with diabetes, low eGFR, or low (< 110 mmHg) standing BP, which might not adequately represent the real-world general population. Moreover, side effects such as orthostatic hypotension were strictly controlled, which also may not represent the real-world conditions. When comparing and interpreting the results of the SPRINT study emphasizing strict blood pressure control with the results in this study, we also would like to mention that the difference in blood pressure measurement environment needs to be considered. In the SPRINT study, researchers adapted Automated Office Blood Pressure (AOBP) method to measure blood pressure. On the other hand, in the past, the method of measuring blood pressure in Korea was a manual measurement method using a mercury sphygmomanometer. In general, manual blood pressure measurements taken directly by medical staff are higher than AOBP blood pressure measurements.

Relatively healthy elderly patients may benefit from low SBP levels in such carefully controlled trial settings, but more caution should be taken in the actual clinical setting. The new ACC/AHA hypertension guidelines revised the target BP for non-institutionalized, community-dwelling elderly from 150/90 to 130/80 mmHg. Our SBP/DBP combination analysis, which has its division points at SBP 130, 150 mmHg and DBP 80, 90 mmHg, may in part predict the potential outcomes of this change in the BP target. For all-cause death, within the same DBP group, the HR was highest when the SBP was < 130 mmHg.

### Strengths and limitations of this study

The present study has its limitations in that only the initial baseline BP was collected for analysis. BP trajectory during the follow-up period could have been observed, which might have led to a deeper understanding of the results of this study. Moreover, although the models were adjusted for major covariates, other confounding factors might remain. A variety of clinical conditions, such as frailty, dementia, malnutrition and dehydration, can affect blood pressure levels in the elderly, so these factors need to be considered when interpreting the results. The fact that these additional blood pressure-related factors could not be considered together due to the retrospective cohort study design is essential when interpreting our results, and this remains a topic of study for future research.

This study had also practical limitations in distinguishing between hemorrhagic stroke due to high blood pressure and intracranial cerebral hemorrhage due to trauma including traffic accidents, only based on health insurance claim data. It should be considered as an important limitation in the interpretation of the results. In addition, ischemic heart disease requiring coronary angioplasty was not considered as a major outcome variable, so it is necessary to consider this point in the design of future studies. Pulse pressure in elderly hypertensive patients is also well-known and important prognostic factor and needs to be considered as an explanatory variable in future studies.

Despite these shortcomings, this study has its strengths. The strengths of this study lie in the nationally representative large population cohort and the validity of the claims data from the NHIS database. This study is also one of the first cohort studies for the very elderly Asian population, which needs to be further studied due to possible racial differences. Moreover, while SBP and DBP have been studied separately in previous reports, their combination has rarely been analyzed. This study provides evidence of the importance of considering both SBP and DBP when managing hypertensive patients. Furthermore, this study indirectly simulates the potential results to be achieved on adapting the revised ACC/AHA target of 130/80 mmHg.

## Conclusions

This may indicate that with regard to mortality, the new target may lead to unwanted outcomes. Moreover, our results show that when DBP is not controlled, a low SBP level does not always guarantee a reduction in morbidity or mortality risk. The association of generally higher HR for outcomes measures with higher DBP within the same SBP level supports that when controlling BP, not only SBP but also DBP need to be taken into consideration. In addition, when SBP exceeded 150 mmHg, the lowest DBPs < 80 mmHg had a higher risk for all three outcome measures compared to DBPs in the range of 80 to 89 mmHg. This signifies that higher pulse pressure, especially when SBP is not controlled, is particularly dangerous for the very elderly population.

This nationwide cohort with a large elderly population evaluated the effects of different BP levels on ischemic stroke, MI, and all-cause mortality hazard. The nadirs, lower subgroups with no significant benefit, and higher subgroups with significant increase in HR implicate measures that might be used in the clinical setting. Further research to validate these results should be carried out in the future to set clear evidence-based standards.

## Data Availability

Data are available from the Korea National Health Insurance Sharing Service Institutional Data Access / Ethics Committee (https://nhiss.nhis.or.kr/bd/ay/bdaya001iv.do) for researchers who meet the criteria for access to confidential data. Researchers can apply for the National Health Insurance data sharing service upon approval of the Institutional Review Board of their institution. After review of the Korea National Health Insurance Sharing Service Institutional Data Access / Ethics Committee, authors are required to pay a data access fee and confirm that other researchers will be able to access the data in the same manner as the authors.
